# Microfluidic Platform with Precisely Controlled Hydrodynamic Parameters and Integrated Features for Generation of Microvortices to Accurately Form and Monitor Biofilms in Flow

**DOI:** 10.1021/acsbiomaterials.4c00101

**Published:** 2024-06-21

**Authors:** Keqing Wen, Anna A. Gorbushina, Karin Schwibbert, Jérémy Bell

**Affiliations:** † Bundesanstalt für Materialforschung und -prüfung (BAM), Unter den Eichen 87, Berlin 12205, Germany; ‡ Freie Universität Berlin, Kaiserswerther Str. 16-18, Berlin 14195, Germany

**Keywords:** E. coli, fluorescence, bacteria trapping, particle velocimetry, topographical pattern

## Abstract

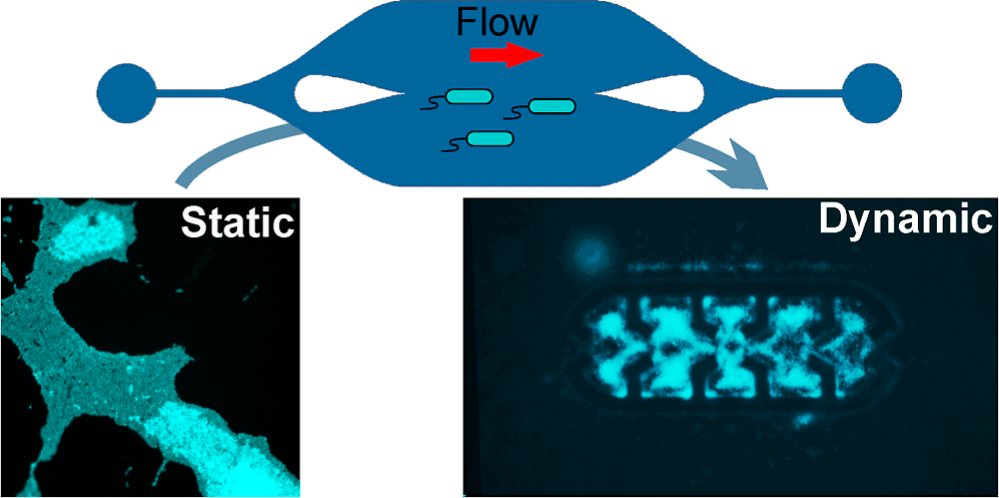

Microorganisms often live in habitats characterized by fluid flow, and their adhesion to surfaces in industrial systems or clinical settings may lead to pipe clogging, microbially influenced corrosion, material deterioration, food spoilage, infections, and human illness. Here, a novel microfluidic platform was developed to investigate biofilm formation under precisely controlled (i) cell concentration, (ii) temperature, and (iii) flow conditions. The developed platform central unit is a single-channel microfluidic flow cell designed to ensure ultrahomogeneous flow and condition in its central area, where features, *e.g.*, with trapping properties, can be incorporated. In comparison to static and macroflow chamber assays for biofilm studies, microfluidic chips allow *in situ* monitoring of biofilm formation under various flow regimes and have better environment control and smaller sample requirements. Flow simulations and experiments with fluorescent particles were used to simulate bacteria flow in the platform cell for calculating flow velocity and direction at the microscale level. The combination of flow analysis and fluorescent strain injection in the cell showed that microtraps placed at the center of the channel were efficient in capturing bacteria at determined positions and to study how flow conditions, especially microvortices, can affect biofilm formation. The microfluidic platform exhibited improved performances in terms of homogeneity and robustness for *in vitro* biofilm formation. We anticipate the presented platform to be suitable for broad, versatile, and high-throughput biofilm studies at the microscale level.

## Introduction

Microorganisms are often found not as single cells but in self-organized communities, known as microbial biofilms. Microbes organized in a biofilm benefit from social interactions, an enhanced rate of nutrient exchange, and an increased tolerance to desiccation and biocides.^1−4^ Thus, appearance of biofilm poses significant risks to industrial or medical systems, especially in wastewater treatment facilities, drinking water distribution systems,^5^ food processing environments, catheters and medical implants,^6^ or bioremediation of oil and gasoline spills. Consequently, a strong interest exists for investigating biofilm, driven by the need to better understand and manage its adverse effects, but also alternatively for biochemical studies on testing of antifouling structures or innovative antibiotic drugs specific to biofilms.^7^ In those cases, the accurate formation of a biofilm at a fast and reproducible rate and position is of utmost importance.

Nevertheless, biofilm formation is a complex process and surface properties such as surface charge density (van der Waals force and electrostatic interactions),^8,9^ stiffness (the ratio of stress to strain),^10,11^ roughness,^12,13^ wettability (water contact angles),^14,15^ and topography^16−19^ have long been recognized as key factors influencing biofilm formation. Current investigation devices such as microwell plates are extensively employed in assessing biofilm formation due to their convenient usage and available instrumentation. More advanced, flow macrodevices show testing flexibilities concerning the shape and size of different material specimens under flow,^20^ but they present a high demand for reagents.^21,22^ It is noteworthy that the dynamic conditions afforded by such devices better correspond to many industrial or medical systems.^23−27^ Dynamic conditions can strongly influence the cell concentration, cell detachment,^28^ and molecular transport regulating the biofilm growth by controlling the availability of nutrients and oxygen.^29^ Therefore, implementation of methods for studies in more natural dynamic environments is a major research direction.^30^ In this regard, microfluidic-based methods are a promising approach in biofilm research.^31^ While several studies in microfluidics focus on single-cell analyses and manipulations, only a few studies concern the in-flow study of biofilms (Section SII, Supporting Information). Those studies are mostly using simple straight channel designs,^32^ without experimental assessment of the flow stabilities or localized inhomogeneities that could influence biofilm formation due to uncontrolled nutrient gradients or inhomogeneous shear forces.^33−35^ In addition, the conditions for biofilm formation may require long incubation time,^36,37^ porous channels with distribution of fluid flow velocities,^38,39^ or some preliminary steps^40^ incompatible with the required high throughput for extensive biofilm studies against a multitude of parameters or for antibiotic testing, for example.

Hence, in this study, a microfluidic platform based on a PDMS microchip was developed to study *Escherichia coli* (*E. coli*) biofilm formation in ultrahomogeneous flow (in terms of velocities and direction) and to correlate this formation with localized microvortices characterized *via* simulations and flow analyses thanks to particle velocimetry techniques.^41^ The investigation of biofilm formation under precisely controlled environments, bacteria concentration, and temperature and under various flow regimes was achieved with a versatile microfluidic platform. With the aid of this platform, some features dedicated to biofilm growth were integrated to obtain rapidly defined, reproducible, and localized biofilms with the aim of getting closer to the concept of digital microfluidics for biofilms.^42^ With such a tool, traditional static or macroscale flow chamber biofilm studies could be easily transformed, allowing for biofilm culture with lower reagent consumption and higher throughput. Moreover, the described method covers some gaps in the existing methods regarding experimental assessment of flows especially regarding the channel’s homogeneity and reproducibility of the biofilm formation. In addition, the simulated and experimental fluidic parameters were correlated with the experimental biofilm growth and distribution.

## Experimental Section

### Microfluidic Assembly

**Figure 1:**
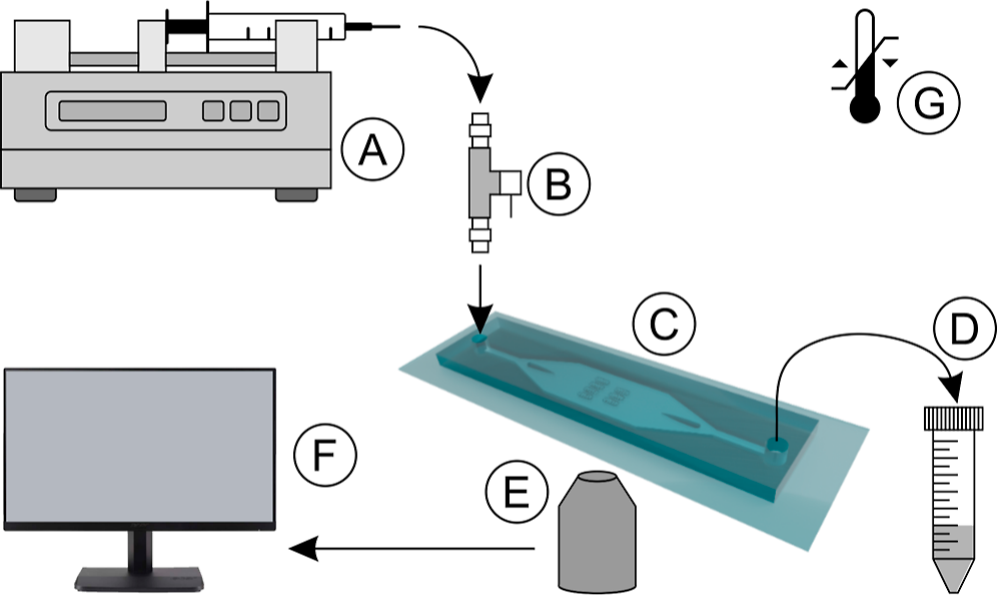
Scheme of the microfluidic-based platform: (A) syringe pump; (B) shut-off valve; (C) microfluidic chip; (D) waste/outflow collecting tube; (E) microscope readout, (F) data analysis, and (G) temperature control.

Injection of the bacteria, medium, and particles was achieved using a syringe pump ensuring flow rate versatility (Figure 1). Additionally, a closing valve was added in front of the chip inlet to fluidically isolate the chip when necessary. Closing the valve ensured the stability of the biofilm structure and mitigated potential disruptions induced by relatively high-pressure changes and backflows upon refilling or exchange of a syringe when injecting a fresh medium after bacterium injection or temporary interruption of flow for the purpose of imaging analysis. The connection between the syringe, valve, microchip, and waste was achieved with 500 μm inner diameter Teflon tubing to increase flow control precision, ensure biocompatibility, and allow for sterilization. The whole miniaturized fluidic platform could be inserted into an incubator for a precisely controlled culture temperature (range 25–80°, temperature stability 0.2°). This temperature range fits the most common temperatures used for biofilm growth and studies. In this study, all biofilm experiments were performed at 28 °C, while focusing mostly on hydrodynamic effects on the biofilm formation.

### Biofilm Formation

The Teflon tubing was steam-sterilized, and all other microfluidic devices were disinfected using 70% ethanol for 15 min, followed by rinsing with sterile deionized water. Microfluidic chips were sterilized with UV radiation for 15 min. An *E. coli* TG1-MRE-Tn7-141 fluorescent strain was plated on LB agar plates containing chloramphenicol (Cm) and gentamycin (Gm) at 15 mg L^–1^ each and incubated at 37 °C overnight. Then, a single colony was picked to inoculate 20 mL of LB medium containing Cm. After overnight incubation at 37 °C and 120 rpm, the culture was diluted 1:100 v/v in fresh, prewarmed LB medium and incubated for an additional 2 h until cells reached an exponential growth phase at an OD_600 nm_ around 0.3–0.8. The culture was then centrifuged for 3 min at 5000*g* and resuspended in minimal medium M9 (3 g L^–1^ KH_2_PO_4_; 6 g L^–1^ Na_2_HPO_4_; 1 g L^–1^ NH_4_Cl; 0.5 g L^–1^ NaCl; 2 g L^–1^ glucose × H_2_O; 0.25 g L^–1^ MnSO_4_ × 7H_2_O; 0.01 g L^–1^ CaCl_2_; pH 7.4) supplemented with thiamine (1 mM) and l-proline (1.7 μM). Then, the bacterial suspension was diluted to an OD_600 nm_ of 0.5 which corresponds to approximately 3 × 10^7^ cfu mL^–1^.

First, the microfluidic device was rinsed with M9 for 5 min at a flow rate of 5 μL min^–1^. Second, the bacterial suspension was injected for 30 min at a flow rate of 0.5 or 3 μL min^–1^. Third, the microfluidic device was connected to a fresh M9 Thi/Pro medium supply which was continuously pumped into the flow cell for 20 h at 28 °C at flow rates of 0.5 or 3 μL min^–1^ throughout the whole experimental procedure.

## Results and Discussion

### Microfluidic Flow Chip Design and Fabrication

**Figure 2:**
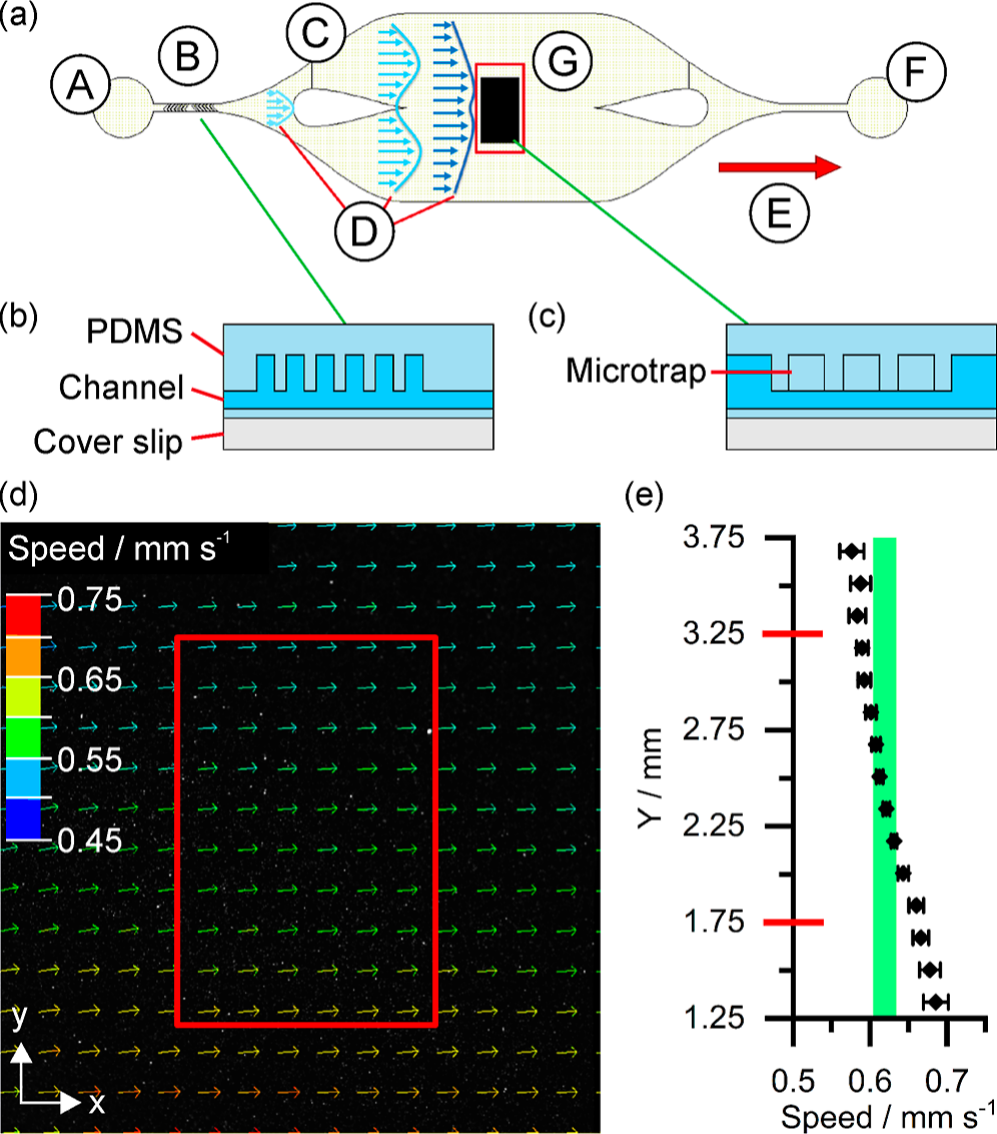
(a) Top-down (XY plan) view of the microfluidic flow cell design: (A) inlet; (B) SHM; (C) dispatcher; (D) flow velocity profiles; (G) central area; (E) flow direction; (F) outlet; cross section view (XZ plan) of the (b) passive mixer and a (c) microtrap feature; (d) vector mapping of the flow velocities at the center of the flow chamber measured from epifluorescence imaging and PIV. (e) Resulting flow velocities [red squares from (a,d) and red marks in the *Y*-axis in (e) correspond to the central analysis area: 1.5 × 1 mm].

Microfluidic devices deal with the manipulation and control of fluid flow at the microscale, for example, the channel internal volume presented in this study is 1 μL. Previous studies showed how the hydrodynamic conditions such as flow velocity inside a microfluidic channel can influence *Pseudomonas aeruginosa* biofilm development.^43,44^ To investigate the relation between microvortices and biofilm formation, avoiding side effects from the inhomogeneous flow (flow rate gradient or multidirectional flows) is necessary. In microfluidic channels, due to the micrometric dimension and the low flow rate, flow is often laminar with a Reynolds number below 1. In this study, the Reynolds numbers calculated for the various tested channel designs ranged from 0.003 to 0.5. The calculated Peclet number for bacteria or nutrients in the water phase ranged from 40 to 1000 indicating limited diffusion mixing. Therefore, to ensure homogeneous injection and flow in a channel dedicated to in-flow biofilm formation, we designed a channel incorporating geometric features for initial solution mixing, flow distribution, and biofilm analysis area free of wall-induced effects or inhomogeneous flows. To ensure uniform bacterium, particle, or nutrient distribution at the entry of the system, a staggered herringbone mixer (SHM)^45−47^ was integrated into the chip design, between the tubing inlet and the main chamber. A microfluidic channel with a 5 mm wide straight channel with a high width-to-height ratio was designed to minimize the wall shear stress effect at the center of the channel while maximizing flow throughput and offering a uniform flow field along a central large analysis area of approximately 1 × 1.5 mm. Two 3 mm long flow distributors were added close to the channel’s inlet and outlet, to generate a smooth and homogeneous linear flow stream distribution in downstream.^48^ Compared with the channel designed by Graham *et al.*,^49^ the design presented here incorporates an SHM and flow distributors for increased homogeneity, rendering it more robust for long-term biofilm studies. Notably, this channel design also incorporated a bubble trap positioned at the inlet of the channel, which is of great interest to prevent bubbles from disturbing the analysis but also has the potential to introduce concentration inhomogeneity within the solution. To account for vortices triggered by features and minimize background interference from bacteria flowing above the feature, the channel height was 15 μm, with the feature height around 10 μm, ensuring a sufficient distance for both factors. According to particle image velocimetry (PIV) analysis results as shown in Figure 2, the central area of this channel presented ultrahomogeneous flow in terms of velocity and direction, enabling potentially highly detailed quantification of flow–biofilm interactions.

The flow chamber design is compatible with various materials used for chip realization; here PDMS was selected, thanks to its high transparency and excellent biocompatibility, ideal for directly monitoring *in situ* biofilm formation (Section SIII, Supporting Information).^50^ For the fabrication of a fully PDMS-based microfluidic channel, including the later features added within the central area, especially because biofilm formation is known to be influenced by surface properties,^51^ a 5 μm-thin PDMS membrane was uniformly coated onto the coverslips after air plasma activation to ensure stability.^52^

### Imaging of Biofilm Formation

Imaging of the process inside the chip was achieved with an inverted epifluorescence microscope and a fluorescent stereo microscope both equipped with a green fluorescent protein filter set. Thanks to their complementarity in terms of resolution and field of view, they allowed the study of full biofilm formation: initial bacterium adhesion, biofilm growth, and in-flow behavior (Section SIV, Supporting Information). In addition to the *E. coli* strain, fluorescent amino formaldehyde polymer microspheres were selected as a model to trace flow pathways. For characterization of flow dynamics inside channels, fluorescent particles have been widely used in microfluidic studies as a model visual tracer, enabling quantitative analysis of flow parameters without the need for biological safety measures or a laboratory. Nonetheless, it should be noted that bacterial motility is not only governed by hydrodynamics. Numerous bacteria are equipped with filamentous cell appendages like flagella and pili, allowing active bacterial locomotion in liquids (swimming motility) and on surfaces (twitching and swarming motility). These cell appendages are also known to act as strong adhesins, facilitating bacteria to firmly attach to surfaces and form a biofilm. Bacteria also exhibit collective behavior. Microbial colonies like biofilm streamers form, influenced by cell–cell communication, in a microfluidic flow.^34,53,54^ The use of model particles is therefore relevant for analyses of flow and vortices in the channel, at the features without bacteria or at the initial stage of bacterial adhesion. The selected particles were bright fluorescent green spherical polymer particles with diameters between 1 and 5 μm, providing intense contrast and visibility relative to background interferences. They could be detected easily by fluorescence microscopy, similar to fluorescent protein-expressing bacteria. While of similar size to *E. coli* TG1, the particle density (1.3 g mL^–1^) slightly exceeded the density of living *E. coli* (1.1 g mL^–1^),^55^ but they could accurately mimic displacement of bacteria in the microchannel (Figure S5). The homogeneous and stable suspension of particles in water is crucial in evaluating the effect of flow; therefore, cetrimonium bromide (1% v/v) was added and the particle suspension (13 g L^–1^) was sonicated for 5 min in an ultrasonic bath. This procedure prevented aggregation and sedimentation for a couple of hours and the suspension could be injected into the microchannel without clogging issues. With excellent fluorescence properties, the suspended polymer microspheres contributed to an improvement of detection sensitivity with a low signal-to-noise ratio making the velocimetry (for low magnification: flows in the channel) or tracking (for high magnification: vortices at the features) analysis easier and more reliable.

### Application

To analyze biofilm formation, a fluorescent mutant strain of *E. coli* TG1 was selected as the test strain. *E. coli* TG1 is known to be a good biofilm former due to its high expression of F-pili.^13^ For easy detection and monitoring of biofilm formation using epifluorescence microscopy, the test strain was equipped with a fluorescent protein tag (Section SVI, Supporting Information).^56^

**Figure 3:**
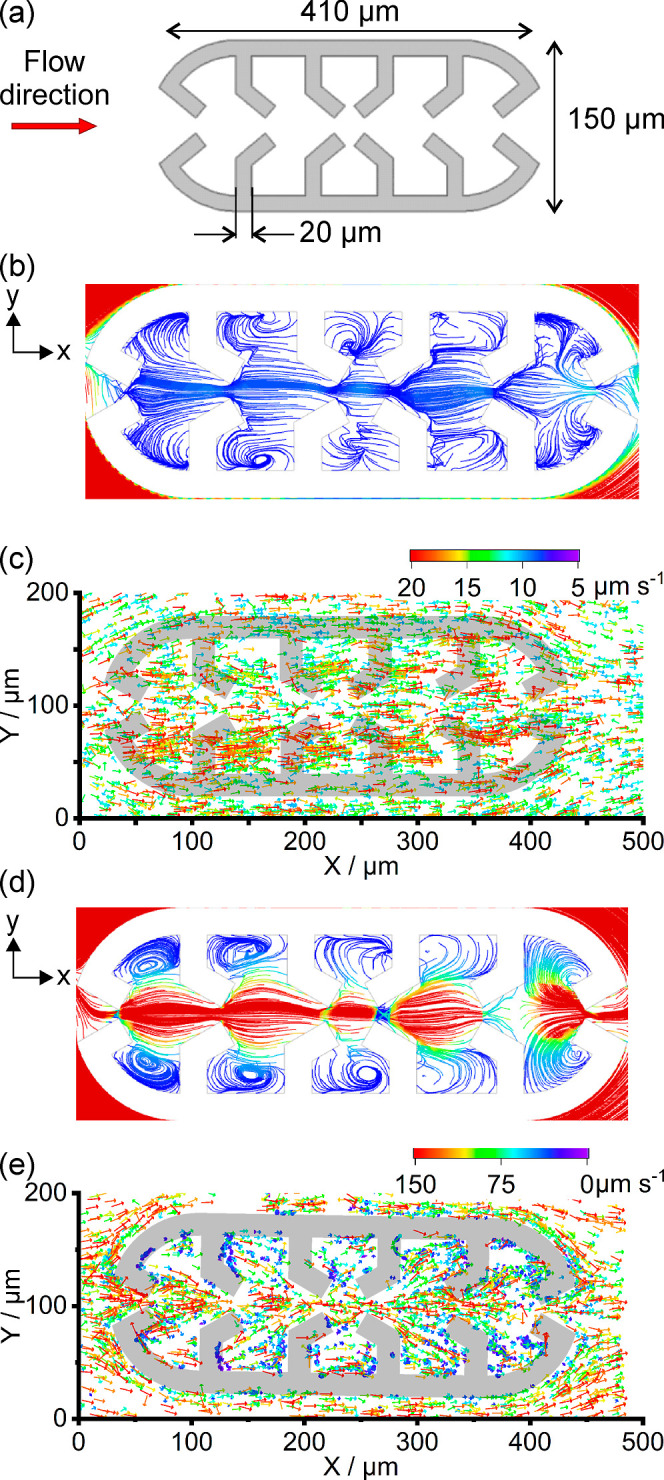
(a) Scheme of the microtrap design with dimensions; flow simulation of water into the microtrap: resulting 2D streamlines inside the microtrap at flow rates of 0.5 (b) and 3.0 μL min^–1^ (d) (rainbow balanced colormap from velocity magnitudes of 0 to 10 μm s^–1^); plot of the experimental movements obtained from PTV analyses of particles above and inside the microtrap at flow rates of 0.5 (c) and 3.0 μL min^–1^ (e).

At the channel central area, where the flows are highly homogeneous, defined microstructures can be integrated easily to induce and investigate localized microvortices and their influence on the biofilm pattern. Two types of scaffolds were integrated, some microtraps or some arrows, and molded in PDMS together with the channel. First, a microstructure defined as a microtrap (7 per channel) was designed, based on a three-dimensional bacterium trap reported by Di Giacomo *et al.*(57) with 3 funnels in flow direction and 3 funnels opposite to flow direction, forming 5 inner cavities. The feature size is 10 μm high, 410 μm long, and 150 μm wide, and the thickness of the wall or ridges is 20 μm as shown in Figure 3a. Such a relatively complex feature is expected to efficiently trap bacteria and favor biofilm formation. Initial simulations showed that such structures should be able to generate microvortices, especially vortices within the microtrap cavities, upon sufficient flow speed (Figure 3b,d). Indeed, it is well-described that before and behind obstacles, flows are forming microscale vortices, the dimensions of which depend on the flow velocities.^58,59^ The successive layers of funnels decreased the outward flux of particles and determined the number of vortices inside the microtrap, which may favor initial bacterial adhesion plus give bacteria a low shear stress environment at different positions to form biofilm. The structures were built to make 2/3rd of the channel height, so the flows can enter the microtrap from the inlet funnel but also from the top, inducing not only two-dimensional vortices but three-dimensional flow displacements (Figures S6 and S7). Calculated flow parameters showed that for a flow rate of 0.5 μL min^–1^, vorticity magnitudes (local spinning motion) and Q criterion (excess rotation rate relative to the strain rate) remained close to zero, but an increase of the flow rate to 3.0 μL min^–1^ is sufficient to increase those values up to 6.7 s^–1^ and 4.1 s^–2^, respectively (Figures S8 and S9). To confirm the simulations, particle velocimetry analyses were performed. At this high magnification, PIV requires a high concentration of nanoparticles, which can lead to clogging; plus, they are not visible with a conventional microscope and do not fit in size to the bacteria being studied. The influence of microtrap geometry on the laminar flow was therefore analyzed by particle tracking velocimetry (PTV) (Figure 3c–e) compatible with low-density medium and moderately distributed particles compared to PIV. The same suspension of fluorescent polymer microspheres described above was used.

The PTV algorithm used the mean flow velocity to estimate an appropriate pixel shift range for the fluorescent particle from successive frames. Typically, a video from the in-flow particles was recorded until approximately 500 frames were recorded (approximately 20 s) and the PTV algorithm was run for all successive 500 frames’ combinations. Two flow rates were tested: 0.5 and 3 μL min^–1^, both compatible with biofilm formation studies. The PTV output showed that upon approaching the microtraps by laminar motility, particle displacement was moderately changed for low flow rates but strongly influenced for a flow rate of 3 μL min^–1^. Due to the microtrap shape, the flow was partly focused at each funnel entry, and upon reaching the microtrap cavities, significant *Y*-direction movements were observed and the particles’ speed strongly decreased. The apparent slowdown of the particles could also be due to their vertical movement, which was not captured in the plane of the recorded video. In this case, three-dimensional PTV would be required to investigate *Z*-axis movement.^60^ The results provided confirmation of trapping particles in flow using such a design.

For biofilm formation, the first step was a prewash with the medium to clean the channels and saturate the materials, to ensure chip-to-chip reproducibility of the glass or PDMS surface properties and hydrophobicity which could strongly influence initial bacterial adhesion.^61^ Second, an *E. coli* TG1-MRE-Tn7-141 suspension with an OD_600 nm_ of 0.5 or 1.0 (3 or 5 × 10^7^ cfu mL^–1^) was injected into the channel for 30 min. This step allowed for initial bacterium adhesion at favorable positions by sedimentation or trapping. After this inoculation period, fresh M9 medium was injected, and the biofilm was cultured overnight from the bacteria present in the channel. Compared with the nutrient-rich medium, M9 does not adsorb on or alter the physicochemical properties of surfaces.^62^ To avoid contamination of the medium supply, a 0.22 μm filter was inserted between the syringe containing fresh medium and the tubing.

**Figure 4:**
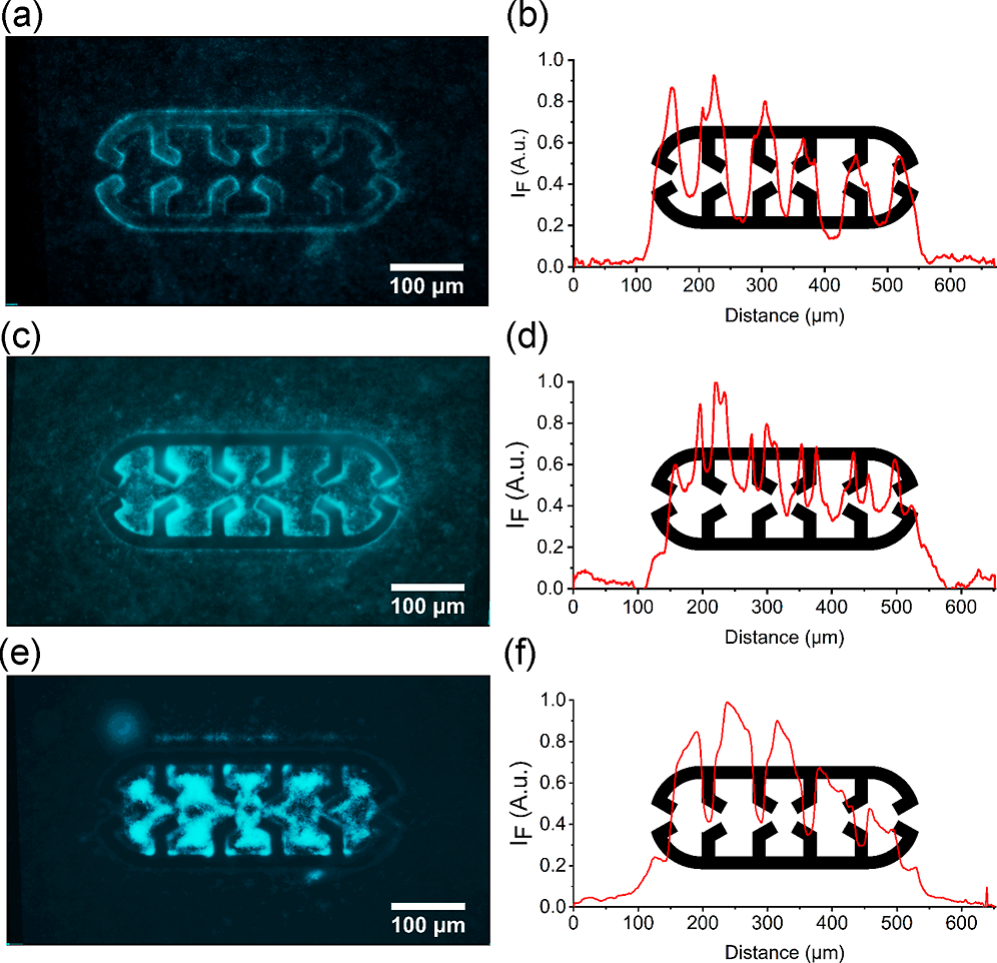
(a) Averaged fluorescence images obtained from images of 7 microtraps and corresponding fluorescence intensity plots of *E. coli* TG1-MRE-Tn7-141 biofilms at the microtrap obtained by extraction of profile intensity with respective initial OD and flow rates of 1.0 and 0.5 μL min^–1^ (a,b); 0.5 and 0.5 μL min^–1^ (c,d); and 0.5 and 3.0 μL min^–1^ (e,f). Flow direction from the left (distance 0 μm) to the right (distance 650 μm).

The ability of the microtrap to capture bacteria and influence biofilm formation was tested at different flow regimes (Figures 4 and S10). Biofilm formation analyses consisted of image treatment (Section SIV, Supporting Information) to obtain for each condition an average image of fluorescent biofilm inside the microtrap and conversion of the image into a fluorescent intensity plot versus X coordinates. In analogy to simulation and PTV analysis results, biofilm formation occurred favorably in the channel’s analysis area at the microtrap. Experiments with a blank channel showed only low and random adhesion of bacteria and no biofilm formation in the tested conditions. For all tested conditions, in the initial phase of bacterium injection, the microtrap walls became predominantly occupied by the bacteria, potentially leading to the appearance of bacteria on the outside walls of the microtrap (Figure S12). Indeed, the trap experiences higher shear forces and vortices at the microtrap ridges.^63^ Upon cultivation, the bacteria residing within the microtrap are more likely to proliferate while being in low-velocity regions with low shear forces (Figure S13) and as a result, biofilm growth appeared strongly dependent not only on initial bacterial adhesion but also on hydrodynamic conditions.^64^

**Figure 5:**
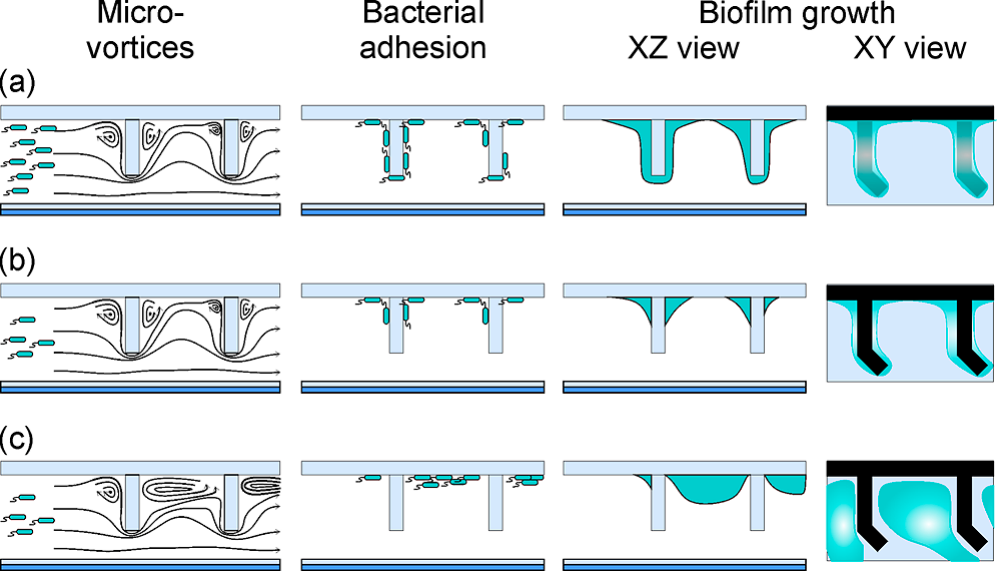
Scheme on hypotheses of microvortices at the microtrap ridges and on *E. coli* TG1-MRE-Tn7-141 adhesion and growth at (a) high bacteria concentration (6 × 10^7^ cfu mL^–1^) and low flow rate (*Re* 0.001); (b) low bacteria concentration (3 × 10^7^ cfu mL^–1^) and low flow rate (*Re* 0.001); and (c) low bacteria concentration (3 × 10^7^ cfu mL^–1^) and high flow rate (*Re* 0.01).

**Figure 6:**
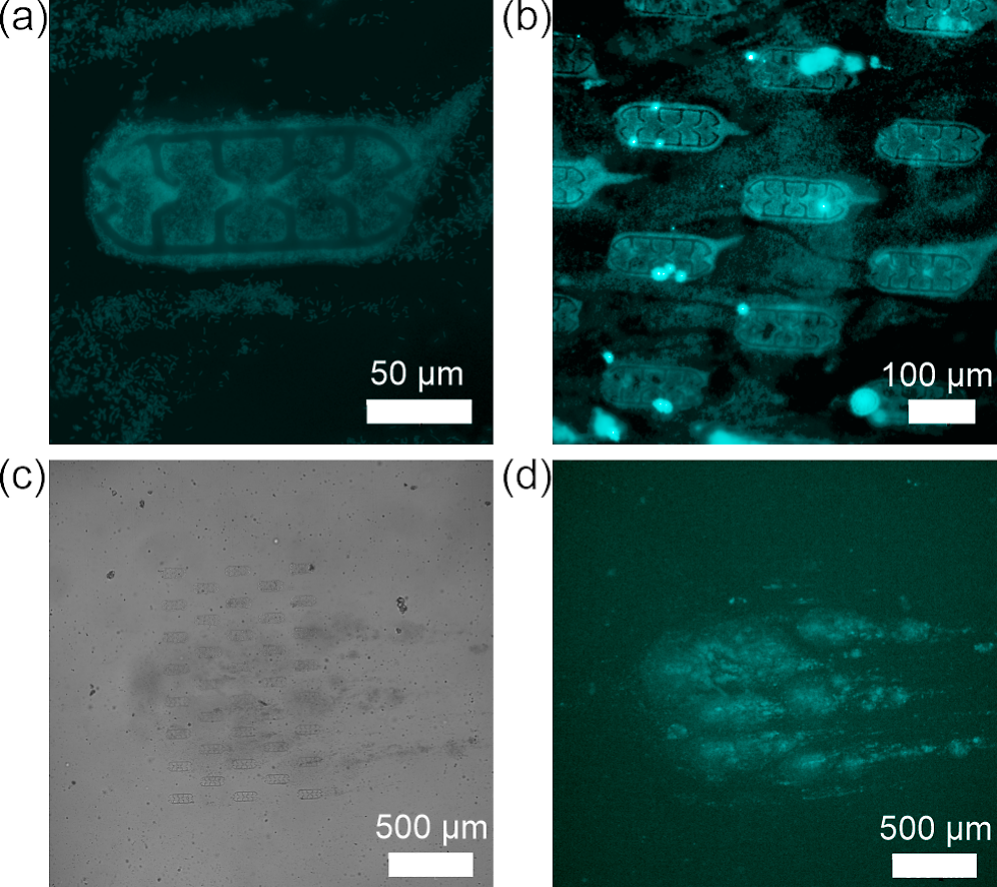
Fluorescence images of slightly overgrown biofilms in a microtrap (a, scale bar 50 μm) and at several microtraps (b, scale bar 100 μm); Brightfield (c) and fluorescence (d) images of highly overgrown *E. coli* TG1-MRE-Tn7-141 biofilms in the flow cell (flow direction from left to right, scale bar 500 μm).

Comparing the results obtained from different initial optical densities (OD), a higher number of bacteria localized on the microtrap walls were observed at an OD of 1.0 (Figure 4a), while bacteria exhibited a preference to remain in the walls’ corners at an OD 0.5 (Figure 4c). Hence, we hypothesize that the high initial concentration of bacteria (Figure 5a,b) led to a greater deposition of bacteria on the walls. Subsequently, during the extended culture period, a larger number of bacteria were observed on the wall rather than inside the trap. The resulting fluorescent plots (Figure 4b,d) showed an enhanced ratio of bacteria on the microtrap walls for an OD of 1.0 compared to an OD of 0.5. The obtained biofilms for the OD of 0.5 and flow rates of 0.5 and 3.0 μL min^–1^ corroborated the increase of microvortices within the trap for increasing flow rate observed for simulations and PTV (Figures 4e and 5c). At low flow rates, the low flow vorticity within the trap (Figure 3b,c) favored the biofilm growth at the walls or in the corners. For higher flow rates, biofilm growth occurred at the cavity centers in accordance with single vortices formed by the microtrap walls (Figure 3d,e), especially the first 3 cavities in terms of flow direction. Such an observation was confirmed on the fluorescence plotted curve with single colonies mostly localized at those 3 cavities (Figure 4f). When the bacteria pass through the first funnel (microtrap inlet), the flow velocity remains sufficiently high, resulting in fewer bacteria getting trapped within the vortices. As the bacteria flow into the second funnel, the flow rate decreases, leading to a larger number of bacteria being retained. This pattern continued with the third funnel. However, after passing through the third funnel, a reduced formation of biofilm compared to the previous funnels was observed, which may be due to availability of nutritional support or bacterium-specific properties and interactions. Such behavior is currently under investigation, studying topographical expression of biofilm-related genes. Furthermore, the flow direction plays a crucial role in the formation of bacterial colonies; as previously described,^65^ it can modulate colonization patterns on surfaces. In accordance, the obtained *E. coli* biofilms showed similar behavior: upon extended biofilm growth (>40 h), biofilms exhibited growth and expansion outside the microtrap, first with appearance of a tail at the side opposite from the flow (Figure 6a,b) and second upon merging of the biofilms from several microtraps. The resulting bigger biofilms self-organized in a comet-like structure (Figure 6c,d) also corresponding to the flow direction.

**Figure 7:**
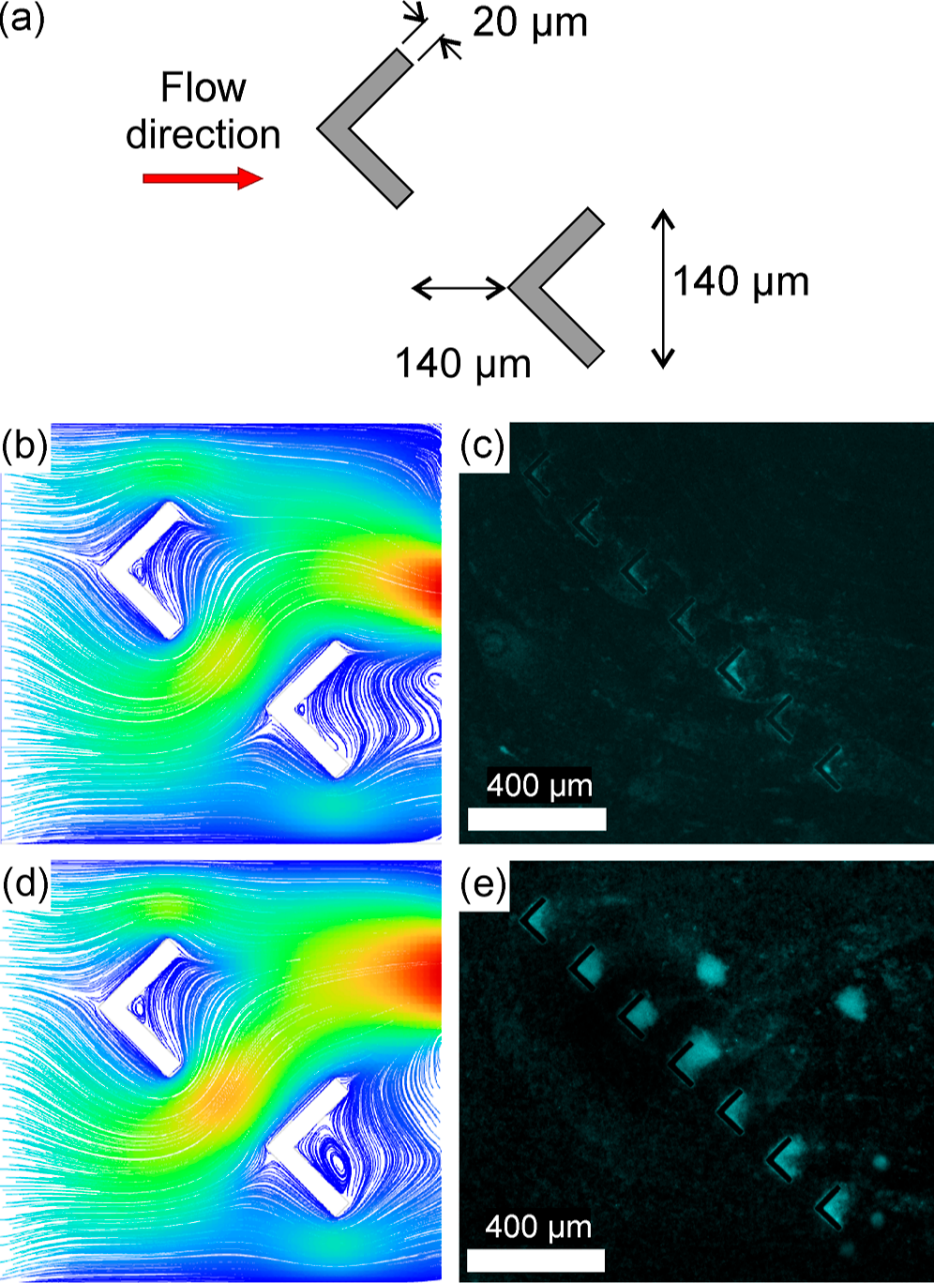
(a) Arrow feature dimensions; simulation of 2D streamlines inside the microtrap at flow rates of 0.5 (b) and 3.0 μL min^–1^ (d) (rainbow balanced colormap from velocity magnitudes of 0 to 200 μm s^–1^); representative fluorescence images of *E. coli* TG1-MRE-Tn7-141 biofilms with an OD of 0.5 and respective flow rates of 0.5 (c) and 3.0 μL min^–1^ (e). Flow direction was from the left to the right.

The second feature design to be tested consisted of a simpler arrow design of 20 × 140 μm (Figure 7a). An ensemble of 7 arrows was placed at the central area of the channel, separated by one arrow distance (140 μm). From the simulation (Figure 7a,b), similar to the microtrap design, upon the increase of the flow rate from 0.5 to 3 μL min^–1^, some microvortices appear in the down-flow area of the features. The PTV analyses were performed using the same suspension of fluorescent polymer microspheres described above (Figure S14). The PTV output showed that upon approaching the arrows by laminar motility, particle displacement was changed for both flow rates. Due to the arrow shape, the flow was partly defocused and refocused upon passing the arrows. At a flow rate of 0.5 μL min^–1^, the downstream flow comes back quickly to unidirectional flow, but at 3.0 μL min^–1^, the downstream flow seems to be disturbed for the rest of the studied window. For biofilm formation, the same protocol as for the microtrap was followed and an *E. coli* TG1-MRE-Tn7-141 suspension with an OD_600 nm_ of 0.5 (3 × 10^7^ cfu mL^–1^) was used. The obtained biofilms for the OD of 0.5 and flow rates of 0.5 and 3.0 μL min^–1^ corroborated the increase of microvortices downstream to the arrows observed for simulations and PTV. At low flow rates, the low flow vorticity (Figure 7b,c) limited biofilm formation just behind the arrow, but for higher flow rates, biofilm formation occurred in the whole cavity formed by the arrows (Figure 7d,e). The interest of such a feature comes first like for the previous design in the spatially controlled and homogeneous formation of a biofilm in hydrodynamic conditions. Second, the flow-focusing stream^62^ induced by the arrow feature potentially induces some gradients of flows and therefore nutrients from the “wings” to the center of the arrows.

Consequently, the developed microfluidic platform proved to be robust and perfectly adapted for biofilm formation under dynamic conditions. Such a platform represents an ideal system for biofilm studies, especially by integrating microtrap or arrow structures affording controlled and homogeneous biofilms and therefore maximizing reproducibility between the experiments and increasing throughput, for example, for biomedical, antibiotic, or microcorrosion studies.

## Conclusions

Here, we designed and established a comprehensive microfluidic system with an ultrahomogeneous flow that enables investigating biofilms under various strictly controlled conditions. The variation of flow can be induced by integrating features for microvortex generation. Some microtrap and arrow structures capable of inducing microvortices were designed, tested, and validated by PTV. To demonstrate the effectiveness and potential of this microfluidic system, we used a fluorescent protein-labeled *E. coli* strain to study the correlation between the hydrodynamic conditions and the biofilm formation. The results indicated that microvortices generated at the microfeatures exerted an influence on the localization and development of biofilm. In conditions of low-velocity flow, the biofilm formation occurred at the corners or on the top of the microstructures. However, with higher flow rates, bacterial deposition and biofilm growth were observed predominantly at the center of the trap or behind the arrows. These results demonstrated the potential of such a microfluidic platform for investigating biofilm formation in a hydrodynamic environment, but furthermore, the robust and controllable biofilm growth at the determined position and of various structures can serve as a valuable tool for the dynamic or *in situ* analysis of biofilms. For example, we anticipate this platform to be an efficient instrument to assess the activity of antimicrobial agents against biofilm formation. Indeed, this dedicated microfluidic platform is versatile and can adapt to different materials, different surface structures, or different surface treatments. Ongoing research focuses on testing the relation between biofilm formation, gene expression, and environmental conditions as well as testing alternative features or patterns and microbial strains.

## Supporting Information Available

Instrumentation and Chemicals, State-of-the-art In-flow Biofilm Formation, Microfluidic Platform, Imaging, Numerical Simulations, Construction and Biofilms of *E. coli* TG1-MRE-Tn7-141, Applications, and Arrow Features (PDF)
