# Supplementary material for: Microfluidic Platform with Precisely Controlled Hydrodynamic Parameters and Integrated Features for Generation of Microvortices to Accurately Form and Monitor Biofilms in Flow
Source: ACS Biomater Sci Eng. 2024 Jun 21;10(7):4626–34. doi: 10.1021/acsbiomaterials.4c00101 (PMC11234330; doi:10.1021/acsbiomaterials.4c00101)
Supplement: Supplementary file 1 — ab4c00101_si_001.pdf [file ab4c00101_si_001.pdf]

Supporting Information

Microfluidic Platform with Precisely Controlled Hydrodynamic Parameters and Integrated Features for Generation of Microvortices to Accurately Form and Monitor Biofilms in Flow

Keqing Wen[†,‡], *Anna A. Gorbushina*[†,‡], *Karin Schwibbert*[†], *and Jérémy Bell*[†]*

[†] Bundesanstalt für Materialforschung und -prüfung (BAM), Unter den Eichen 87, Berlin 12205, Germany

[‡] Freie Universität Berlin, Kaiserswerther Str. 16−18, Berlin 14195, Germany

*Email: jeremy.bell@bam.de

I. Instrumentation and Chemicals S2

II. State-of-the-art of In-flow Biofilm Formation S3

III. Microfluidic Platform S4

IV. Imaging S5

V. Numerical Simulations S8

VI. Construction and Biofilms of *E. coli* TG1-MRE-Tn7-141 S10

VII. Applications S12

VIII. Arrow Features S14

IX. References S15

I. Instrumentation and Chemicals

Climatic chamber Innova 44 (New Brunswick Scientific, Edison, USA) was used to provide the controlled environment for the cultivation of bacteria and biofilm formation. Optical density was checked under 600 nm, with Ultrospec 10 cell density meter (Biochrom Ltd., Cambridge, UK). The obtained OD showed a good linearity regarding Colony Forming Units (CFU) counts per mL: $\frac{CFU}{mL} = OD_{600\,nm} \times 5.6 \times 10^7$ (R^2 = 0.97). For precise and controlled injection of bacterial suspensions and particle solutions, Fusion 100 syringe pumps (KR Analytical Ltd, Sandbach, UK) were employed. The DMi8 inverted fluorescence microscope with objective Leica HC PL APO 20x/0.75 IMM CORR CS2 (Leica Microsystems GmbH, Wetzlar, Germany) was used to take fluorescent images of bacteria with GFP filter set. SXZ16 Olympus Stereo microscope (Olympus Life and Material Science Europa GmbH, Hamburg, Germany) was employed to record videos for particle imaging and tracking analysis. PIV and PTV analysis were completed by using MicroVec software (Microvec Pte Ltd., Singapore).

All materials and chemicals were used as purchased. Spherical fluorescent amino formaldehyde polymer particle (FMG - Green Fluorescent Polymer Microspheres, Cospheric LLC, Santa Barbara, USA) of 1.3 g cc^{-1} density, median diameter of 1.5 – 2 µm, and emission at 515 nm were purchased for PIV and PTV.

II. State-of-the-art of In-flow Biofilm Formation

Ref.	Year	Platform	Channel ($l \times w \times d$ mm)	Flow characterization	Strain	Application	Biofilm formation time
1	2019	Microfluidics	T-junction (1.5) × 0.1 × 0.02	*n.r.*	*T. marina*	Effects of flow velocity and nutrient concentration on biofilm	≥ 6 days
2	2019	Microfluidics	Custom/gradient 35 × 20 × 2	Simulations	*S. oneidensis, C. testosteroni*	Biofilm development under defined solute gradients	≥ 7 days
3	2020	Microfluidics	Flow focusing 30 × 3 × 0.18	*n.r.*	*E. coli*	Culture media influence on bacterial colonization, antibiotic eradication of sessile bacteria	≥ 60 h
4	2022	Centrifugation /Microfluidics	Growth chamber 4 × 0.4 × 0.1	Simulations	*E. coli*	Dynamics of antibiotic resistance selection in bacterial biofilms	≥ 6 days
5	2012	Microfluidics	Straight + feature *n.r.* × 0.57 × 0.09	Simulations	*E. coli*	Formation of biofilm streamers in vortical flow	30 min
6	2018	Annular reactor	*n.a.*	*n.r.*	*Wild biofilm*	Effect of flow conditions on biofilm morphology and growth in drinking water	≥ 4 weeks
7	2017	Macro-flow chamber	*n.a.*	*n.r.*	*L pneumophila*	Biofilm removal in water supply systems	≥ 70 h
8	2015	FC 71 Flow Cell	Straight 25 × 6 × 2	Simulations	*L pneumophila*	Simulation of drinking water biofilm formation	≥ 29 weeks
9	2014	Microfluidics	Straight 23 × 13 × 0.24	*n.r.*	*P aeruginosa*	Biofilm growth and morphology response to chemical gradients	≥ 3 days
10	2012	Microfluidics	Porous channel 3.75 × 0.625 × 0.05	Simulations	*P. fluorescens*	Biofilm formation in a mimicking porous media	≥ 16 h
11	2023	Microfluidics	Porous channel 22 × 4.05 × 0.1	Simulations	*B. subtilis*	Biofilm formation on porous medium to study biofilm permeability	24 h
12	2023	Microfluidics	Straight + feature 5 × 0.4 × 0.06	Simulations	*P. putida*	Development of early-stage biofilm	14 h
this study	-	Microfluidics	Straight + features 5 × 5 × 0.015	Simulations, PIV, PTV	*E. coli*	Correlation of biofilm adhesion and growth with microvortices	20 h

n.r. = not reported; n.a. = not applicable

III. Microfluidic Platform

Microfluidic Flow Chip Design and Fabrication

The microfluidic cell was designed on a vectorial design software, LayoutEditor (juspertor, Unterhaching, Germany). A silicon wafer was used as substrate to produce the mold for the microfluidic cell. First, the silicon wafer was cleaned with acetone and dried at 200°C for 2 min on a hot plate C-MAG HP 4 (IKA, Staufen, Germany). Surface activation was achieved in air plasma (Zepto, Diener Electronic, Ebhausen, Germany) at 0.6 mbar for 2 min. An epoxy-based negative photoresist SU-8 (Kayaku Advanced Materials, Westborough, MA, USA) was spin-coated onto the wafer using a spin-coater POLOS SPIN150i (SPS, Putten, Netherlands). The spinning speed was set first at 500 rpm for 10 s with an acceleration of 100 rpm s^{-1} to spread the photoresist. Then it was increased to 5250 rpm s^{-1} for 30 s to get a 15 µm thick layer. After standard soft baking, a maskless aligner (MLA100, Heidelberg Instruments, Heidelberg, Germany) was used for photoresist UV irradiation at a power of 460 mJ cm^{-2}. The wafer was post baked and immersed in mp6 developer (Kayaku Advanced Materials, Westborough, MA, USA) for 4 min to remove the excess of resist. After microscopic control, the mold was finally baked at 180°C for 2h.

PDMS monomers and curing agent (Sylgard 184, Dow Corning, Midland, USA) were mixed in a 10:1 ratio by weight, stirred, sonicated for 5 min, and placed into a desiccator under vacuum for 20 min to remove bubbles. The mixture was then cast onto the mold and cured at 140°C for 15 minutes. After crosslinking, the PDMS was carefully peeled off from the mold. The excess of PDMS was cut and 1.5 mm inlets and outlets holes were punched for tubing insertion.

A thin PDMS membrane on the cover glass was spin coated using 1 mL of PDMS mixture, poured on the precleaned coverslip. A 5000 rpm s^{-1} spin speed hold for 5 min was used to get a 5 µm thick PDMS membrane which could be cured at 140°C for 2 minutes.[13] This layer of PDMS over galls avoids inequal bacterial adhesion between the two type of surfaces.[14] Specifically, under identical flow conditions, the number of adhered bacteria on the glass surface was five times higher compared to the PDMS-coated glass (Figure S3).

The PDMS layer and PDMS coated 24 × 60 mm glass cover slip (Marienfeld, Lauda-Königshofen, Germany) were both activated with air plasma at 0.6 mbar for 100 s, and then bound together to afford a perfectly sealed chip.

IV. Imaging

Unless stated, the flow direction on all the plots, pictures or simulation is from the left to the right. Imaging of bacterium adhesion and biofilm formation process inside the chip was achieved with an inverted epifluorescence microscope thanks to high resolution and suitable fluorescent illumination for cell counting with Green Fluorescent Protein (GFP) filter set with dichroic filter cutting wavelength at 460nm. The objective lens (20x; Working distance: 0.66 mm; Numerical Aperture: 0.75) used for counting detection afforded a field of view of 666 x 666 µm. Using standard 1 mm microscope glass slide as sealing of the PDMS chip was not possible due to the limited working distance, therefore, it was replaced by 24 x 60 mm cover slip with thickness of 170 µm. The high-quality fluorescent images and Z-stack imaging could be realized to calculate bacterium coverage area in the chip and to check bacteria and biofilm preferential 3-dimensional position (Figure S8). While the objectives adapted to epi-fluorescence microscopy are providing high-magnification and small field of view, they allow to focus (i.e., in flow) only on a fraction of the bacteria flowing in the channel in the chip (Figure S5). Therefore, the obtained movies or series of pictures showed important noise coming from out of focus bacteria and could not be exploited for PIV or PTV analyses. Hence, another type of microscopy for in-flow analysis was used. For this, a fluorescent stereo microscope was selected, using a large diameter objective (1x, Working Distance: 81mm; Numerical Aperture: 0.15; Field of view: 3.5mm) with a large field of view, enabling focusing on all moving particles inside the microfluidic chamber in the vertical axis as shown in Figure S5. Flow imaging was recorded with a USB CCD Camera at 30 frames s^{-1} in bright-field and fluorescence modes (GFP filter set) to monitor the vortices occurring in the chip and especially at the analysis area, through PIV or PTV analyses with the MicroVec software. The lower maximum magnification of this stereo microscope did not allow for bacterium precise localization and counting, thus both microscopy techniques must be used in complementarity to study both bacterium adhesion, biofilm growth and in-flow behavior.

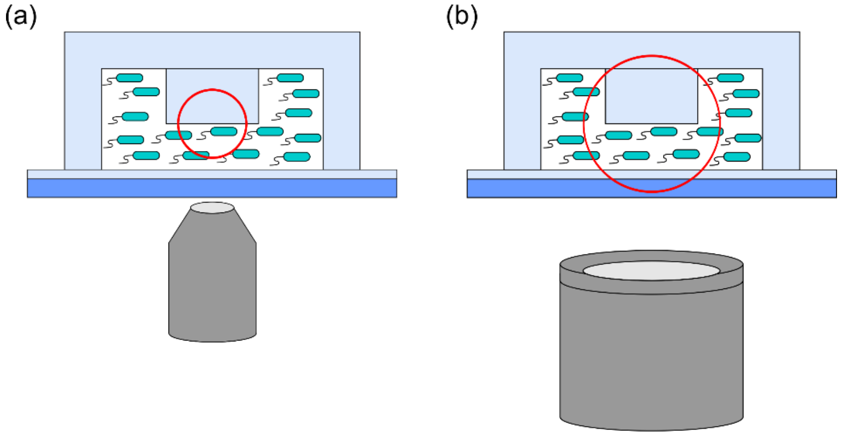

Figure S1. Comparison of different working and parfocal distance of objectives: (a) Standard microscope objective; (b) Stereomicroscope objective.

For each condition tested, characterization of resulting biofilm was achieved by epifluorescence microscopy. For every condition tested, i.e. OD 1.0 and 3 μL min^{-1}, 3 repetitions were performed. For each repetition, a chip includes 7 microtraps, making a total of 3 × 7 pictures (Figure S6).

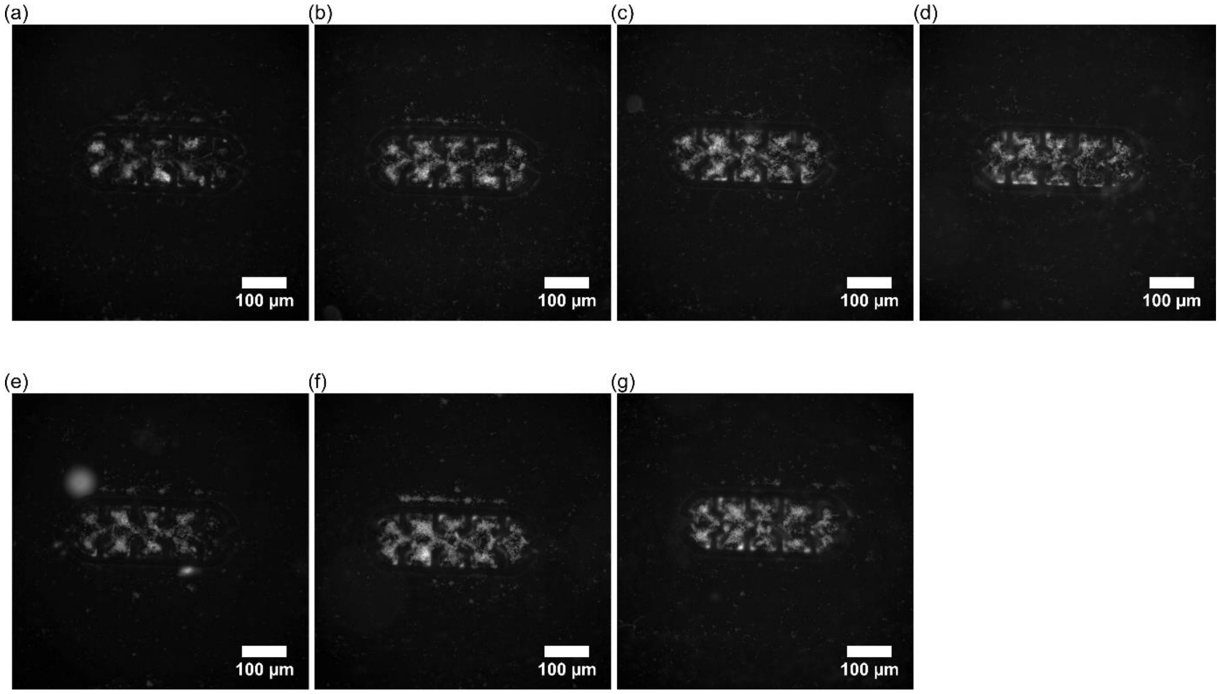

Figure S2. Example of the seven images captured from 1 chip for OD 0.5 and flow rate of 3 μL min^{-1}.

Image treatment consisted first in aligning them and adjust their rotation and dimensions with CorelDRAW software (Alludo, Ottawa, Canada). Then using ImageJ software (National Institutes of Health, Bethesda, USA) including Plugin Grouped_ZProjector (Holly Mountain Software, Wilkesboro, USA), an average picture of each repetition was compiled (Figure S7).

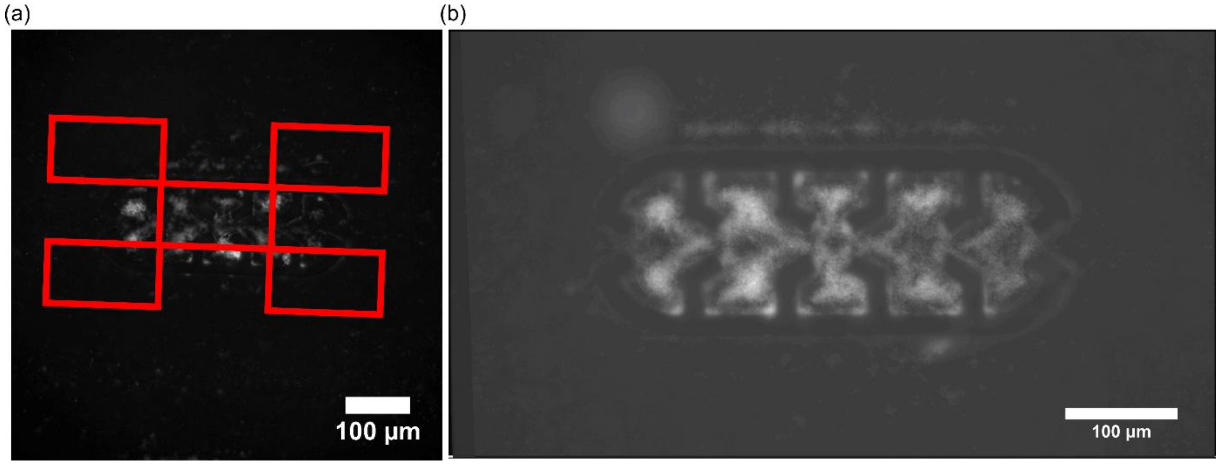

Figure S3. (a) The rotation and dimensions of single images were adjusted using preset masks (red squares); (b) Compilation of an average image.

The biofilm fluorescent intensity along flow direction was extracted using ImageJ profile function. Complete image profile was extracted, as well as the profile from area outside of the microtrap for baseline correction by simple subtraction. The resulting profiles from the three repetitions were averaged and plotted using OriginLab (OriginLab Corporation, Northampton, USA). For illustrative purposes, the averaged images were also corrected in CorelPHOTO using brightness, contrast, saturation, shadows and midtones parameters (Figure S8).

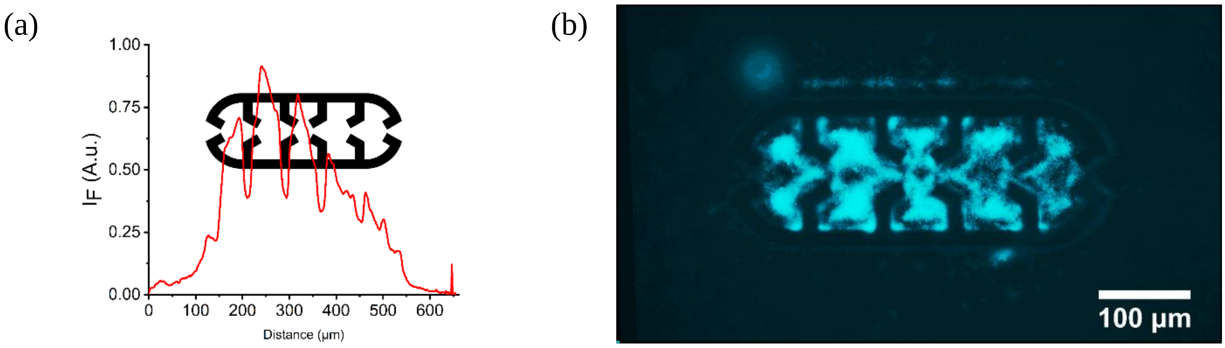

Figure S4. (a) Fluorescent intensity plot along flow direction obtained from ImageJ profile function; (b) Illustrative fluorescent image after corrections (brightness and colors).

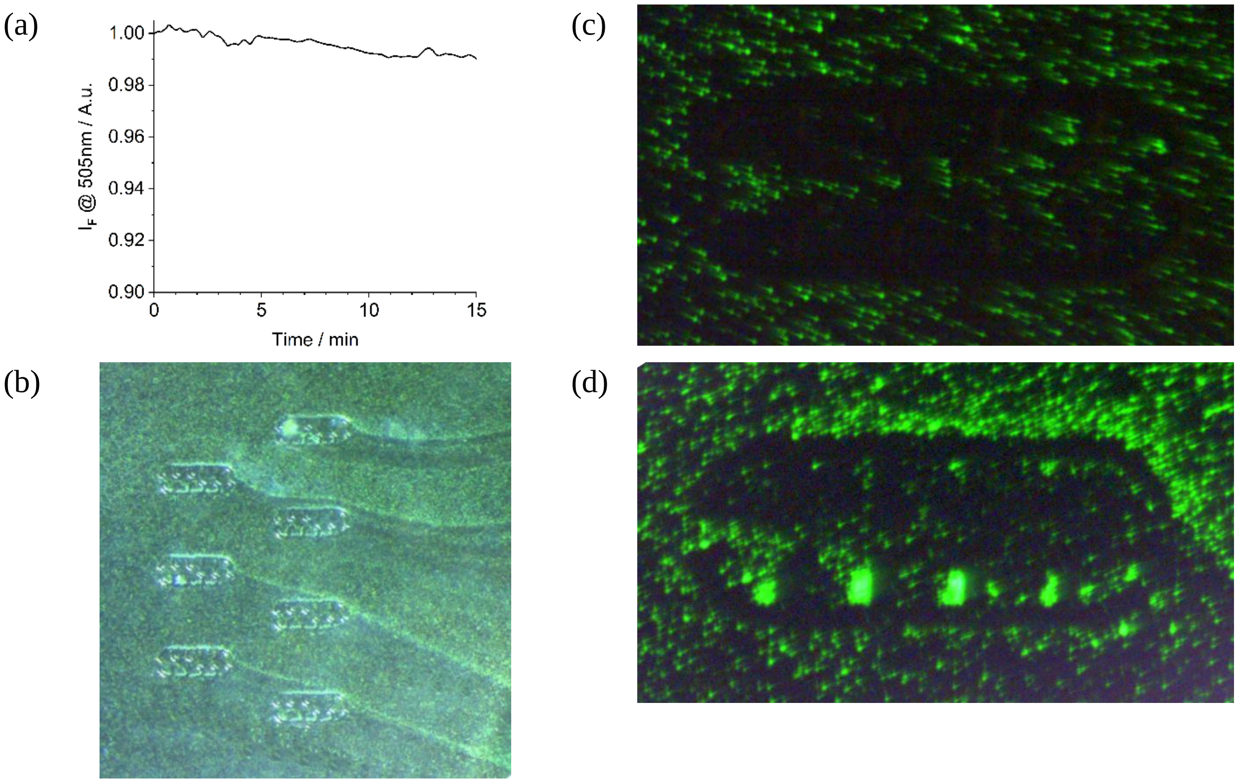

Figure S5. (a) Emission intensity of the particle suspension in water in presence of CTAB over time; (b) Picture in brightfield mode of the channel's central area upon flowing of fluorescent microspheres for PIV or PTV. Pictures in fluorescent mode of a microtrap upon flowing of fluorescent microspheres at the initial injection time (c) and after few minutes (d).

V. Numerical Simulations

Numerical simulations were conducted to characterize the flow vortices inside the microtrap. The geometry of a single microtrap was recreated in FreeCAD. The geometry was exported to ANSYS 2023 R1 (ANSYS Inc., USA), meshed and imported into Fluent module to solve the differential equations governing the balance of mass, momentum, and transport of species. The flow was considered laminar due to its low Reynolds number. Pressure–velocity terms were coupled using k-omega Shear Stress Transport (SST) model. Second-order upwind scheme was used for discretization of convective terms. Boundary conditions included preset flow rates at the inlets, no-slip at the walls of the channels and microtrap and ambient pressure at the outlet.

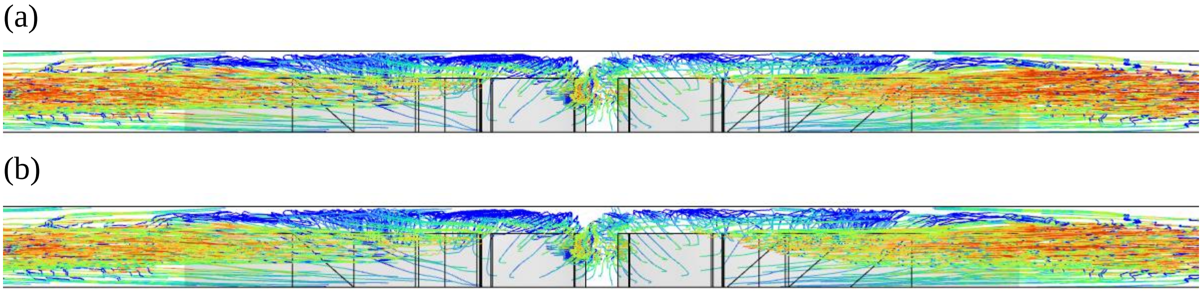

Figure S6. Simulations of flow path lines (YZ plan, view from inlet) with rainbow balanced color mapping of the velocity magnitude: (a) 0.5 µL min^{-1} (color map: 0 to 10 µm s^{-1}) and (b) 3.0 µL min^{-1} (color map: 0 to 50 µm s^{-1}).

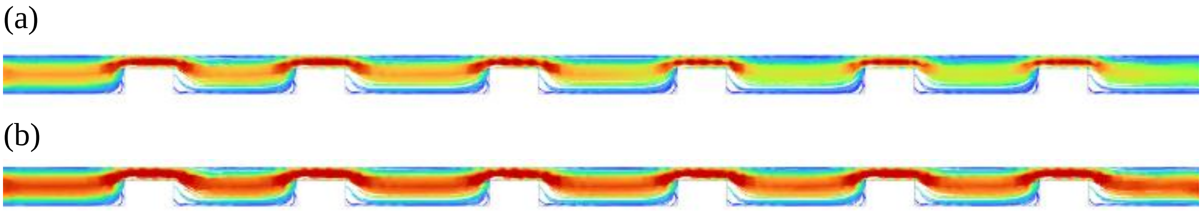

Figure S7. Simulations of flow path lines (XZ plan, flow from left to right) with rainbow balanced color mapping of velocity magnitude: (a) 0.5 µL min^{-1} (color map: 0 to 200 µm s^{-1}) and (b) 3.0 µL min^{-1} (color map: 0 to 1000 µm s^{-1}).

(a)

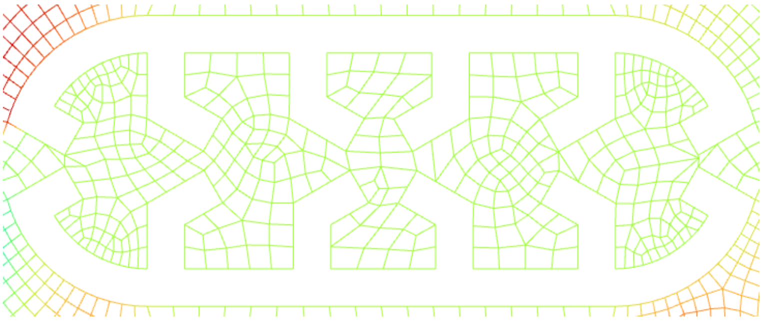

(b)

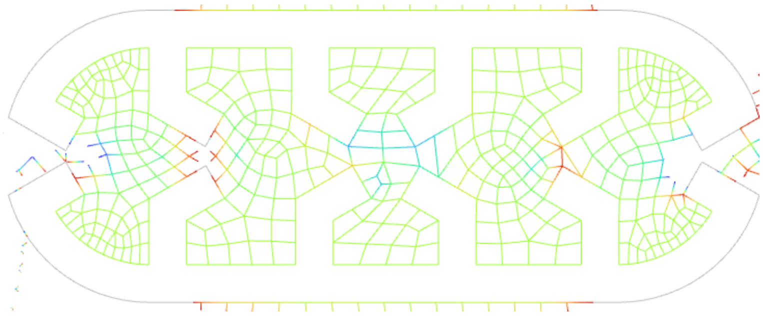

Figure S8. Simulation of Q-criteria inside a microtrap (XY plan, flow from left to right) at flow rates of (a) 0.5 μL min^{-1} and (b) 3.0 μL min^{-1} with rainbow balanced color mapping from –5 to 5 s^{-2}.

(a)

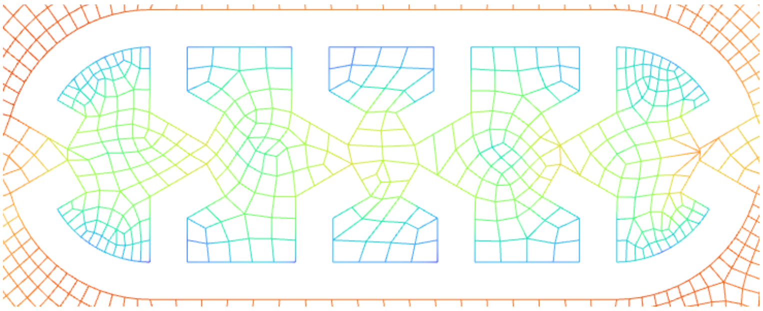

(b)

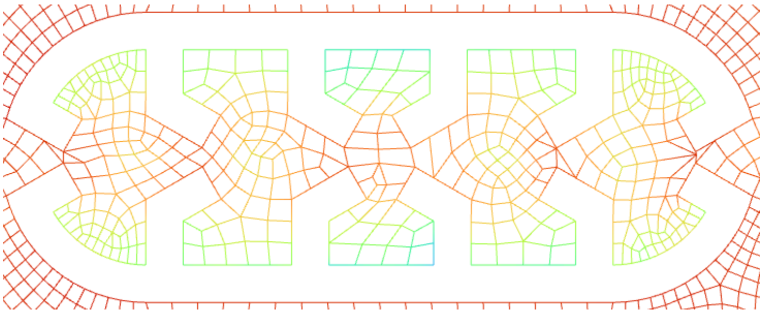

Figure S9. Simulation of vorticity magnitudes inside a microtrap (XY plan, flow from left to right) at flow rates of (a) 0.5 μL min^{-1} and (b) 3.0 μL min^{-1} with rainbow balanced color mapping from 0 to 50 s^{-1}.

VI. Construction and Biofilms of E. coli TG1-MRE-Tn7-141

For biofilm experiments and easy detection and monitoring of biofilm formation using epifluorescence microscopy, *Escherichia coli* TG1 (DSM6056) was fluorescently labelled according to the procedure described by *Schlechter* and *Remus-Emsermann* 2019 with minor modifications.[15]

E. coli S17-1 carrying the Tn7 transposon delivery plasmid pMRE-Tn7-141 was obtained from Addgene.[16, 17] This plasmid is mobilizable, contains Tn7 transposon and a temperature-sensitive origin of replication which does not allow replication above 32°C. In addition, a gene encoding for the mTurquoise2 fluorescent protein, and genes that confer resistances against ampicillin, chloramphenicol, and gentamymcin are present on the plasmid.

With *E. coli* S17-1 as helper strain, plasmid pMRE-Tn7-141 was transferred to *E. coli* TG1 via conjugation. Recipient strain *E. coli* TG1 was grown on Lysogeny broth (LB) (10 g L^{-1} Tryptone; 5 g L^{-1} Yeast extract; 10 g L^{-1} NaCl; 20 g L^{-1} Agar) agar media for overnight at 37°C. Freshly grown overnight single colony was used to inoculate a liquid culture in LB medium. After overnight incubation (37°C, 120 rpm) the culture was diluted 1:100 v/v with fresh medium and further incubated until exponential growth was reached ($OD_{600\ nm}$ 0.8). 10 mL. Donor strain *E. coli* S17-1pMRE-Tn7-141 was grown overnight at 28°C on LB agar plates supplemented with ampicillin (100 mg L^{-1}) to select for the plasmid. Freshly grown overnight single colony was used to inoculate a liquid culture in LB medium plus Amp 100 mg L^{-1}. After overnight incubation (28°C, 120 rpm) the culture was diluted 1:100 v/v with fresh medium and further incubated until exponential growth was reached ($OD_{600\ nm}$ 0.5) 10 mL culture were centrifuged (2000 g, 5 min) and resuspended in 5 mL 1× phosphate buffered saline (1×PBS)(80 g L^{-1} NaCl; 2.4 g L^{-1} KCl; 14.2 g L^{-1} Na_2HPO_4; 2.4 g L^{-1} KH_2PO_4).

10 mL recipient strain and donor strain were mixed 1:1 v/v, then centrifuged (2000 g, 5 min) and resuspended in 100 µL 1×PBS. Bacterial mixes were dropped on MM63 agar plates (MM63) (13.6 g L^{-1} KH_2PO_4; 4.2 g L^{-1} KOH; 2.0 g L^{-1} $(NH_4)_2SO_4$; 0.25 g L^{-1} $MnSO_4$ x $7H_2O$; 1.1 mg L^{-1} $MnSO_4$ x $7H_2O$; 5 g L^{-1} glucose x H_2O; 20 g L^{-1} agar; pH 7.2) containing chloramphenicol (15 mg L^{-1}) and gentamycin (15 mg L^{-1}) and incubated over night at 28°C. LB liquid medium with $0.1\%_w$ arabinose, and Amp (100 mg L^{-1}) was inoculated with single colonies of *E. coli* TG1 pMRE-Tn7-141. After overnight incubation (28°C, 120 rpm), the culture was diluted 1:100 in fresh medium and further incubated for 12–16h. Then, the culture was diluted in 1×PBS to 10^{-6}, plated onto LB plates and cultured at elevated temperature (37°C) to supress propagation of the plasmid. 10 or more individual colonies were picked onto LB agar plates with and without Amp 100. The colonies that did grow on the LB plate, but not on the LB plus Amp, were verified with epi-fluorescent microscopy for the presence of the fluorescent protein tag.

As shown in Figure S10, the genetic modification resulted in a strain exhibiting a bright and stable fluorescence at 475 nm in biofilms. Moreover, these fluorescent strains enabled not only analysis of bacterial adhesion and biofilm formation, but also dynamic behavior when injected into an in-flow microfluidic platform coupled to real-time visualization, allowing further understanding of in-flow bacterial adhesion, proliferation, and release event over time.[18]

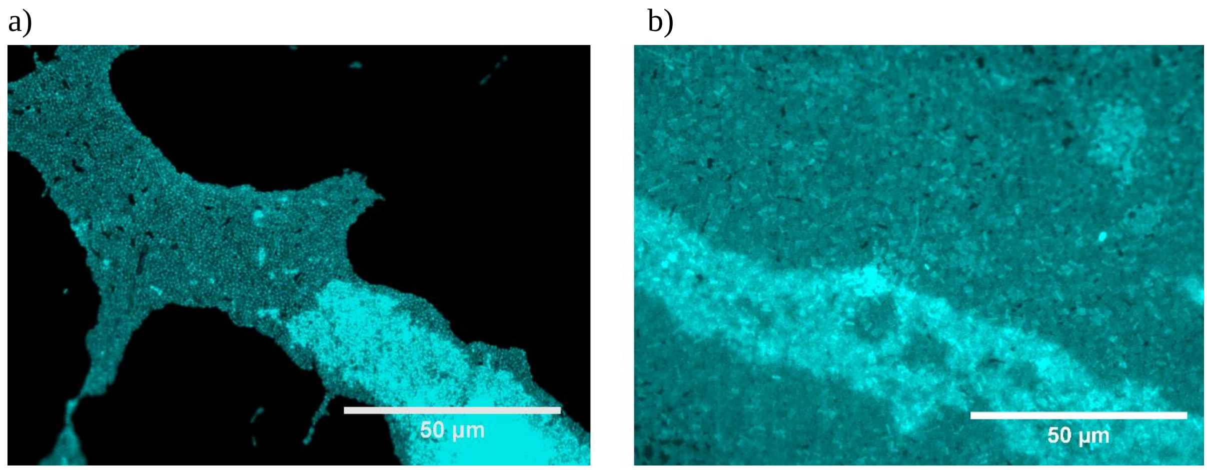

Figure S10. Epifluorescence microscopy images of *E. coli* TG1-MRE-Tn7-141 biofilm formation on glass slides after 3 hours (a) and 21 hours (b) static incubation in a microwell plate with M9 minimal medium. λ_{exc} = 435 nm, λ_{em} = 475 nm.

VII. Applications

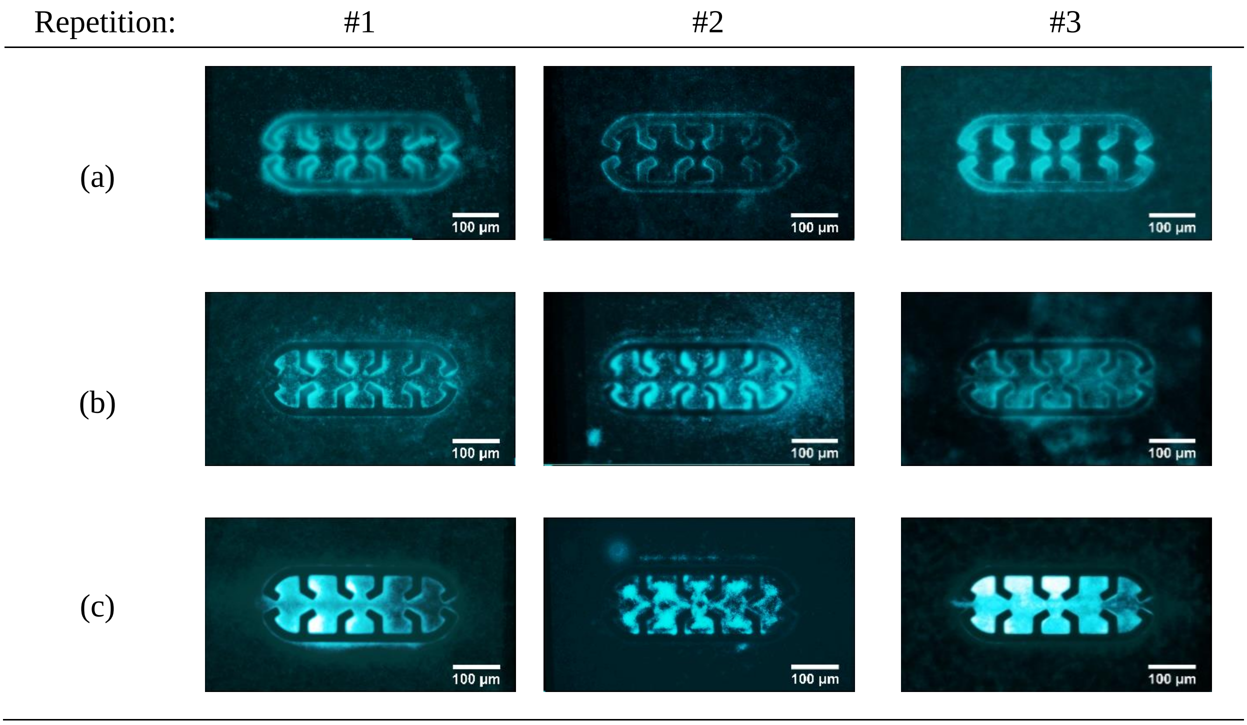

Figure S11. Averaged fluorescence images of the obtained *E. coli* TG1-MRE-Tn7-141 biofilms at the microtrap under different conditions respectively initial OD and flow rates of 1.0 and 0.5 µL min^{-1} (a); 0.5 and 0.5 µL min^{-1} (b); 0.5 and 3.0 µL min^{-1} (c).

The robustness of the microfluidic platform allowed for formation of almost identical biofilms at each microtrap (Figure S2) within one experiment. In addition, the repetition for identical parameters (temperature, flow rate, strain, nutrients) even on different days showed excellent reproducibility and a marginal number of outliers was observed.

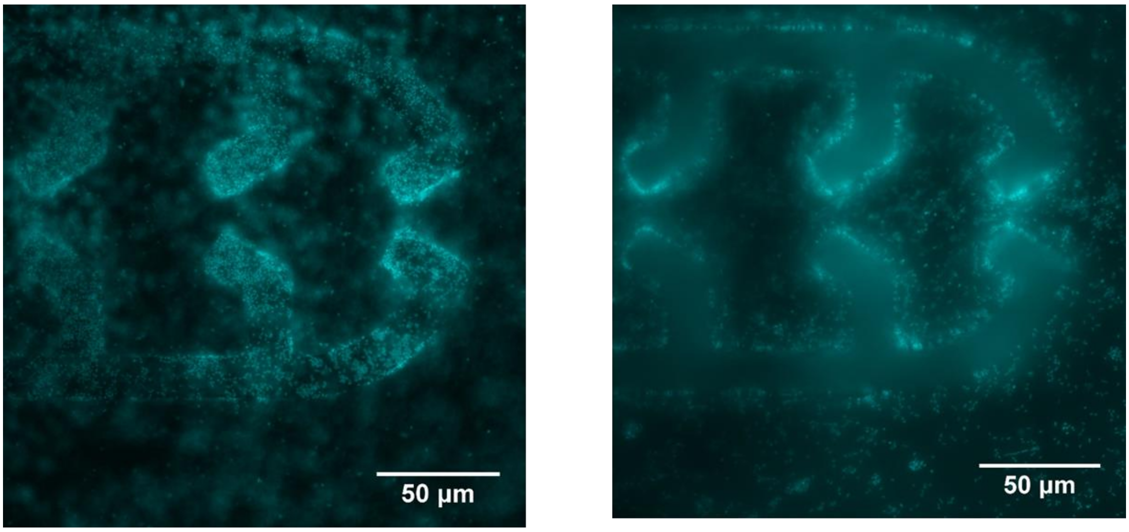

Figure S12. Fluorescence images of the obtained *E. coli* TG1-MRE-Tn7-141 biofilms at the microtrap at different z staking positions.

(a)

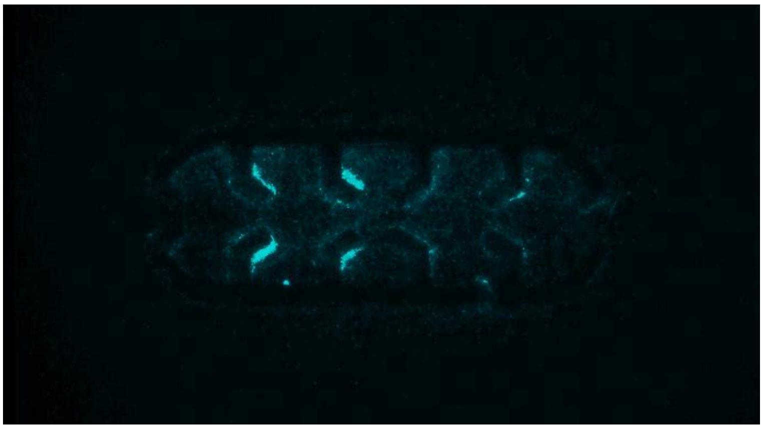

(b)

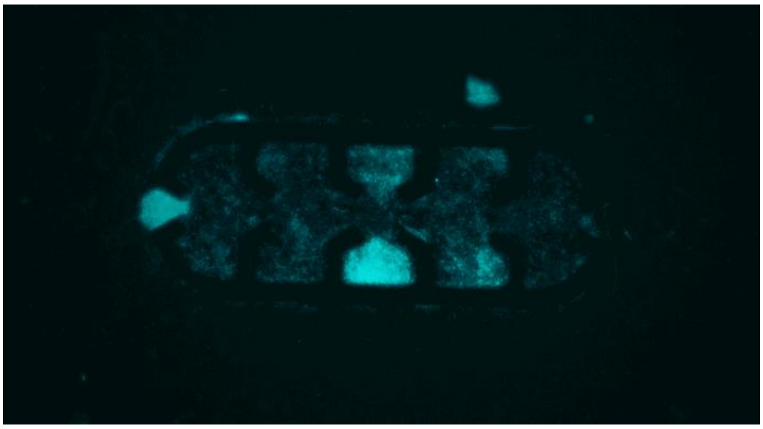

Figure S13. Averaged fluorescence images of the obtained *E. coli* TG1-MRE-Tn7-141 biofilms at the microtrap with an initial OD of 0.5 and a flow rate of 3.0 µL min^{-1} (a) after initial injection of the bacterium suspension (1h) and (b) after 10h of experiment.

VIII. Arrow Features

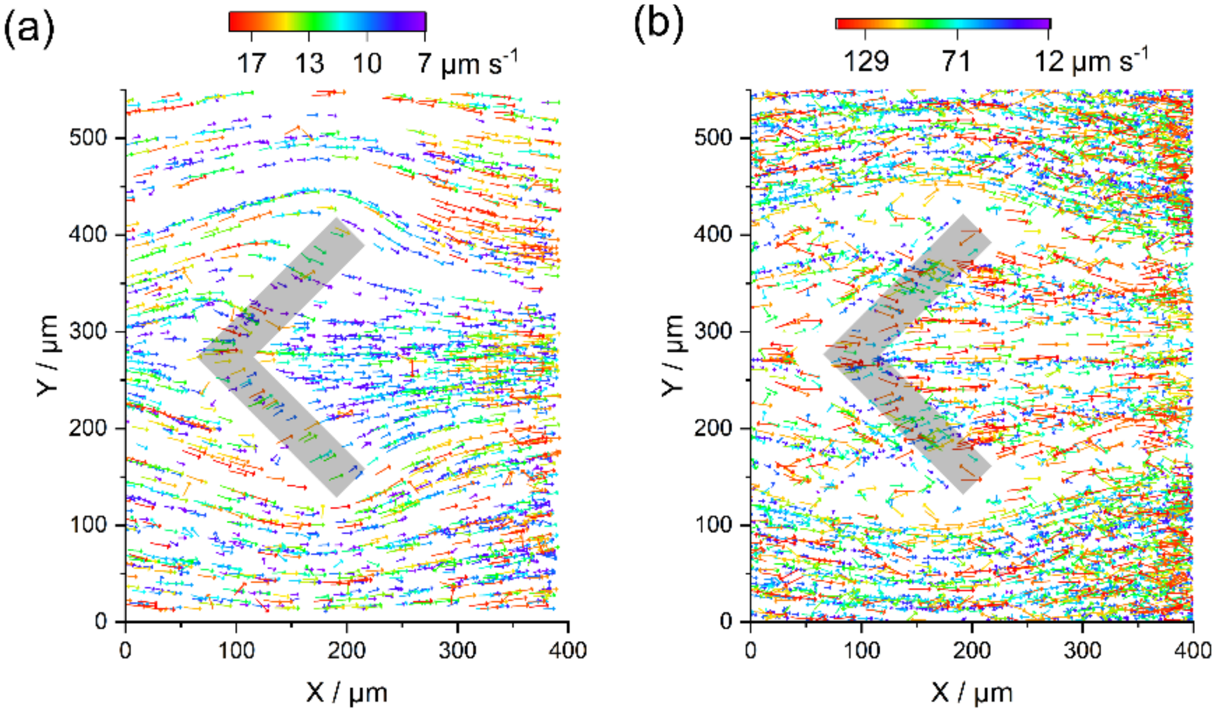

Figure S13. Experimental movements of particles around the arrow features at flow rates of 0.5 μL min^{-1} (a) and 3.0 μL min^{-1} (b) obtained from PTV analyses.

IX. References

1. Liu, N.; Skauge, T.; Landa-Marban, D.; Hovland, B.; Thorbjornsen, B.; Radu, F. A.; Vik, B. F.; Baumann, T.; Bodtker, G. Microfluidic study of effects of flow velocity and nutrient concentration on biofilm accumulation and adhesive strength in the flowing and no-flowing microchannels. *J. Ind. Microbiol. Biotechnol.* **2019**, *46*, 855–868.
2. Zhang, Y.; Li, C.; Wu, Y.; Zhang, Y.; Zhou, Z.; Cao, B. A microfluidic gradient mixer-flow chamber as a new tool to study biofilm development under defined solute gradients. *Biotechnol. Bioeng.* **2019**, *116*, 54–64.
3. Straub, H.; Eberl, L.; Zinn, M.; Rossi, R. M.; Maniura-Weber K.; Ren, Q. A microfluidic platform for in situ investigation of biofilm formation and its treatment under controlled conditions. *J. Nanobiotechnology* **2020**, *18*, 166.
4. Tang, P. C.; Eriksson, O.; Sjogren, J.; Fatsis-Kavalopoulos, N.; Kreuger, J.; Andersson, D. I. A Microfluidic Chip for Studies of the Dynamics of Antibiotic Resistance Selection in Bacterial Biofilms. *Front. Cell. Infect. Microbiol.* **2022**, *12*, 896149.
5. Yazdi, S.; Ardekani, A. M. Bacterial aggregation and biofilm formation in a vortical flow. *Biomicrofluidics* **2012**, *6*, 44114.
6. Tsagkari, E.; Sloan, W. T. Turbulence accelerates the growth of drinking water biofilms. *Bioprocess Biosyst. Eng.* **2018**, *41*, 757–770.
7. Oder, M.; Fink, R.; Bohinc, K.; Torkar, K. G. The influence of shear stress on the adhesion capacity of Legionella pneumophila. *Arh. Hig. Rada. Toksikol.* **2017**, *68*, 109–115.
8. Shen, Y.; Monroy, G. L.; Derlon, N.; Janjaroen, D.; Huang, C.; Morgenroth, E.; Boppart, S. A.; Ashbolt, N. J.; Liu, W. T.; Nguyen, T. H. Role of biofilm roughness and hydrodynamic conditions in Legionella pneumophila adhesion to and detachment from simulated drinking water biofilms. *Environ. Sci. Technol.* **2015**, *49*, 4274–4282.
9. Song, J. L.; Au, K. H.; Huynh, K. T.; Packman, A. I., Biofilm responses to smooth flow fields and chemical gradients in novel microfluidic flow cells. *Biotechnol. Bioeng*. **2014**, *111*, 597–607.
10. Valiei, A.; Kumar, A.; Mukherjee, P. P.; Liu, Y.; Thundat, T. A web of streamers: biofilm formation in a porous microfluidic device. *Lab Chip* **2012**, *12*, 5133–5137.
11. Kurz, D. L.; Secchi, E.; Stocker, R.; Jimenez-Martinez, J. Morphogenesis of Biofilms in Porous Media and Control on Hydrodynamics. *Environ. Sci. Technol.* **2023**, *57*, 5666–5677.
12. Wei, G.; Yang, J. Q. Impacts of hydrodynamic conditions and microscale surface roughness on the critical shear stress to develop and thickness of early-stage Pseudomonas putida biofilms. *Biotechnol. Bioeng.* **2023**, *120*, 1797–1808.
13. Elveflow. *How to make a spin-coated PDMS layer?* https://www.elveflow.com/microfluidic-reviews/soft-lithography-microfabrication/pdms-membrane-thickness-of-a-spin-coated-pdms-layer/ (accessed 06, 2024).
14. Graham, M. V.; Mosier, A. P.; Kiehl, T. R.; Kaloyeros, A. E.; Cady, N. C. Development of antifouling surfaces to reduce bacterial attachment. *Soft Matter* **2013**, *9*, 6235–6244.
15. Schlechter, R. O.; Remus-Emsermann, M. N. Delivering "Chromatic Bacteria" Fluorescent Protein Tags to Proteobacteria Using Conjugation. *Bio Protoc.* **2019**, *9*, e3199.

16. Schlechter, R. O.; Jun, H.; Bernach, M.; Oso, S.; Boyd, E.; Munoz-Lintz, D. A.; Dobson, R. C. J.; Remus, D. M.; Remus-Emsermann, M. N. P. Chromatic Bacteria - A Broad Host-Range Plasmid and Chromosomal Insertion Toolbox for Fluorescent Protein Expression in Bacteria. *Front. Microbiol.* **2018**, *9*, 3052.
17. *pMRE-Tn7-141 was a gift from Mitja Remus-Emsermann (Addgene plasmid # 118557; http://n2t.net/addgene:118557; RRID:Addgene_118557)*.
18. Zhao, X.; Illing, R.; Ruelens, P.; Bachmann, M.; Cuniberti, G.; de Visser, J.; Baraban, L. Coexistence of fluorescent Escherichia coli strains in millifluidic droplet reactors. *Lab Chip* **2021**, *21*, 1492–1502
